# Novel API Coated Catheter Removes Amyloid-β from Plasma of Patients with Alzheimer’s Disease

**DOI:** 10.24966/and-9608/100055

**Published:** 2021-10-06

**Authors:** Rishi Raj Chhipa, Omkar Gandbhir, Hao Le, Sadhana Sankar, Pazhani Sundaram

**Affiliations:** Recombinant Technologies LLC, 1090 Meriden Waterbury Turnpike, Suite 1, Cheshire, Connecticut 06410, USA

**Keywords:** Amyloid, Alzheimer Disease, Alzheimer’s Therapy, Plasmapheresis, Amyloid beta-Peptides, Amytrapper catheter, Extracorporeal device, Amyloid removal

## Abstract

Alzheimer’s disease (AD) is the most common cause of dementia, characterized by the deposition of Amyloid-beta (Aβ) plaques in the brain. We have previously developed Amytrap peptide (the active pharmacological ingredient, API) and linked it to a sepharose bead matrix by click chemistry to form Amytrapper matrix, which was able to bind and remove Aβ from human sera and plasma spiked with biotinylated Aβ42 (bio-Aβ42) *in vitro*. To extend the logic of the previous studies, the current study investigates whether the Amytrap peptide coated inside a medically viable polycarbonate catheter (Amytrapper catheter) could bind and retain Aβ from the human sera. The Amytrapper matrix and the novel Amytrapper catheter were able to bind and retain spiked bio-Aβ42 from human sera or native Aβ from plasma of AD patients. Additional characteristics of the Amytrapper catheter are evaluated and presented in this study. The results presented here provide a proof-of-principle for the first time that extracorporeal Amytrapper device aids clearance of native Aβ (from plasma of AD patients). Thus, our device Amytrapper, either in the form of Sepharose matrix or catheter, could become a novel therapeutic strategy to remove Aβ from circulation in AD patients.

## Introduction

Amyloid-beta (Aβ) plays an important role in the onset and progression of Alzheimer’s disease (AD). The significant presence of extracellular accumulation of Aβ in the brain is the hallmark of AD pathology [1]. Many strategies aim for AD therapy via peripheral Aβ clearance. Despite attempts to develop novel strategies to enhance the removal of Aβ, clinical trials of small molecule pharmacotherapy and immunotherapies to reduce brain Aβ burden have not shown efficacy [2–4]. Plasma exchange (PE) with albumin replacement has been recently developed and clinical trial data showed some benefit for mild and moderate AD patients [5,6]. However, PE would remove other critical substances from patients’ plasma. The removal of plasma Aβ could also be achieved by *ex vivo* adsorption methods wherein anti-Aβ antibodies, albumin, sLRP1 or other proteins [3,7–9] were used as ligand on a solid matrix to remove Aβ from the plasma. However, the adsorption methods could be expensive or associated with danger of infection and low effectiveness [10,11]. Thus, we propose a more specific and safer apheresis model with no or limited side effects. We aimed to generate an amyloid-β trapper (Amytrapper) by immobilizing the retro-inverso peptide (RI-peptide) in a catheter-based plasmapheresis approach to deplete circulating Aβ from the plasma of AD patients.

We have developed and studied Amytrap peptide, a retro inverso peptide (RI peptide: WKGEWTGR) that has been shown to sequester Aβ in both *in vitro* and *in vivo* studies [12,13]. The RI-peptide has a high binding affinity for Aβ. RI peptides are generally non-toxic, non-immunogenic, stable and offer higher resistance to proteolytic degradation [12]. In addition, non-immune based therapeutics are expected to circumvent the immunological side-effects. Based on its Aβ binding affinity, RI-peptide was chosen for incorporation into our Amytrapper extracorporeal system [13]. Amytrapper would be utilized for removal of Aβ, thus reducing the Aβ burden within the AD patient. Amytrapper could handle the circulating levels of Aβ in the range of 350–400 pg/ml plasma of AD patients. The approximate Aβ binding capacity, based on theoretical estimation, is at least 10 times the circulating levels of Aβ [13].

In our previous study, we designed Amytrapper matrix as an apheresis device and demonstrated proof of concept *in vitro*. The design of the device included a plasma separator to separate plasma and blood cells and a column of Amytrapper matrix, which has been described [13]. In this current study, we described an extension of the Amytrapper technology to simplify the process of Aβ removal in peripheral circulation. In this system, a medical catheter coated with the pegylated RI peptide replaces the matrix column. In the present study, we demonstrate in vitro binding, by the catheter, of native Aβ from plasma of AD patients for the first time. In addition, we demonstrate the capture of spiked Aβ42 from normal human blood by Amytrapper catheter. We further evaluated the specificity of this binding. The results show encouraging binding pattern that warrant further investigation to make the catheter a beneficial extracorporeal device to deplete circulating amyloid in many relevant diseases. The ultimate goal is to use this device as a disease modifying agent to gain clinical benefits in the patients.

## Methods

### Generation and confirmation of RI-Mal-PEG4 conjugated peptide

Tetramic RI peptide (MW 4793) was synthesized by Lifetein (Somerset, NJ). Conjugation of Tetramic RI peptide and Mal-PEG4 was performed by Biosynthesis (Lewisville, Texas). The conjugation was purified and assayed using LC-MS by Biosynthesis. Successful conjugation was confirmed using ELISA for detection of tetramer peptide levels in the conjugate. On a microplate, different concentrations of conjugated peptide (0, 1, 5, 10 ng) were added in triplicate and incubated for 1 h at 37°C. The microwells were then blocked with 3% gelatin in TBS for 1h at 37°C. Wells were probed with rabbit anti-RI-peptide antibody [12] at 1/2500 in TBST for 1h at 37°C, then washed 10 times with TBST. The wells were treated with goat anti-rabbit HRP antibody (1/5000, Invitrogen) for 1h at 37°C. After washing the wells 10 times with TBST, 100 μl HRP substrate solution (SureBlue, KPL, Gaithersburg, MD, USA) was added and incubated for 1min at RT. One hundred microliters of 1N HCl was added to stop the reaction and the absorbance was read on Elx800 plate reader at 450nm.

### ELISA of RI-Mal-PEG4 conjugated peptide binding to Aβ42

Binding activity of the tetramer RI-Mal-PEG4 conjugated peptide to Aβ42 was tested by ELISA. Microplate wells (Grainer bio-one) were coated with 100ng of the tetramer RI-Mal-PEG4 conjugated peptide in carbonate buffer pH 9.6 for 1 h at 37°C. Wells were blocked with 3% gelatin in Tris-buffered saline (TBS) for 1 h at 37°C. Different concentrations (25, 50, and 100 ng) of bio-Aβ42 (AnaSpec, CA, USA) was added in triplicate and incubated for 1h at 37°C. The microwells were washed 5 times with TBST and incubated with streptavidin-HRP (1: 5000, KPL, Gaithersburg, MD) for 1 h at 37°C. After washing the wells 5 times with TBST, 100 μl HRP substrate solution (SureBlue, KPL, Gaithersburg, MD, USA) was added and incubated for 1 min at RT. One hundred microliters of 1 N HCl was added to stop the reaction and the absorbance was read on Elx800 plate reader at 450nm.

### Generation of RI-peptide (Amytrap peptide) catheter

Conjugated RI peptide was coated on the polycarbonate catheter by Formacoat (Chaska, MN) by their proprietary technology. Briefly, the catheter was treated by utilizing 3 steps. The catheter surface was treated with low pressure ionized gas (plasma). The crosslinker and PEG Linker were mixed and introduced to the surface of the polycarbonate tube, followed by the exposure of crosslinker and PEG conjugated RI Peptide. All Aβ binding and flow through evaluations were performed on the catheter tubes (50 mm in length with 3.175 mm internal diameter).

### Evaluation of Aβ binding efficiency of the RI-peptide on catheter

This was performed using PBS and human serum treated with bio-Aβ42 and plasma from AD patients. Amytrapper catheter (plus API catheter), or uncoated catheter (minus API catheter) were blocked with 0.01% BSA in PBS (Sigma) overnight at 4^0^C. BSA was removed and catheters were washed 5 times with PBS. The catheters were incubated with 40 ng of bio-Aβ42 in 350 μl PBS or 40 ng of bio-Aβ42 in 350 μl human normal serum (Innovative Research, MI) or 350 μl plasma from AD patients (Innovative Research, MI). After 2 hours incubation at room temperature, sample solutions were transferred to Elisa plate for measurement of bio-Aβ42 or Aβ. Catheters were washed with PBS for 10 times. Catheters were incubated with mouse monoclonal anti-β-Amyloid 6E10 antibody (1/2000, BioLegend) for 1 hour at room temperature. Catheters were washed 10 times with PBS and then incubated with goat anti-mouse HRP (Invitrogen) for 1 hour at room temperature. Catheters were washed for 10 times with PBS. Catheters were incubated with 350 μl of SureBlue TMB substrate (KPL) for 2 minutes at room temperature. Substrate was collected in an Eppendorf tube. 350 μl of 1N HCl was added to stop the reaction. 100 μl of mixture was transferred to a 96 well clear plate and read at 450 nm on the Elx800 plate reader.

### Evaluation of Aβ binding efficiency of the RI-peptide on catheter using whole blood

To assess bio-Aβ42 binding efficiency from whole blood spiked with bio-Aβ42, we used heparinized human blood (Innovative Research Inc., MI) and spiked it with bio-Aβ42. Briefly, catheters were initially blocked with 3% gelatin in Tris-buffered saline (TBS) for 1 h at 37°C. Whole blood spiked with 100 ng/ml of bio-Aβ42 was passed through the catheters at a flow rate of 250 μl/min with a peristaltic pump. After passage, catheters were washed 5 times with TBST and incubated with streptavidin-AP (1: 10000, KPL, Gaithersburg, MD) for 1h at 37°C. After washing the catheters with TBST, 350 μl Lumi-Phos 530 chemiluminescent solution (Lumigen, MI, USA) was added as substrate and incubated for 1 min at RT. The luminescence was read on Biotek Synergy HTX multi-mode reader.

### Evaluation of Aβ binding efficiency of Amytrapper matrix on Alzheimer’s patient plasma

One hundred microliters of Amytrapper matrix (Sepharose beads plus API) was transferred into a clean Eppendorf tube. Unconjugated matrix (Sepharose beads minus API) served as negative controls. 350 microliters of human AD patient plasma (Innovation Research, MI) were incubated with matrices for 30 minutes at room temperature. Supernatants were transferred to clean tubes for measurement of native Aβ. Matrices were washed 5 times with 1 ml TBST. At the end of the fifth wash, the matrix was made up to a volume of 1ml from which 200*μ*l from each tube (in triplicate) was transferred to clean tubes and- incubated with anti-mouse Beta Amyloid antibody 6E10 (1:2,000, BioLegend) for 1 h at room temperature. Beads were washed 7 times with 1 ml TBST and incubated with 1 mL of goat anti mouse HRP for 1 hour at room temperature (1:10,000, Invitrogen). Matrices were washed in 1mL TBST for 10 times and incubated with 50*μ*l TMB substrate (SeraCare, MD) for 5 min and color reaction was stopped with 50*μ*l of 1N HCl. Supernatants were transferred to a microplate and read at 450 nm.

### Measurement of flowthrough bio-Aβ42 by ELISA

50 μl of pre- or post-catheter and pre- or post-column fractions each was mixed with 50 μl of bicarbonate buffer (pH 9.6) and transferred to microplate wells for 1h at 37°C for binding. Wells were then blocked with 3% gelatin in Tris-buffered saline (TBS) for 1 h at 37°C. Plates were washed 3 times with TBST and incubated with streptavidin-HRP (1: 5000) (KPL, Gaithersburg, MD) for 1 h at 37°C. After washing the wells 3 times with TBST, 100 μl HRP substrate solution (SureBlue, KPL, Gaithersburg, MD, USA) was added and incubated for 1min at RT. One hundred μl of 1N HCl was added to stop the reaction and the absorbance was read at 450nm.

### Specificity of Amytrapper catheter

Amytrapper catheter’s specificity for its target molecule, Aβ42, was analyzed by incubating unlabelled Aβ42 through Amytrapper catheter and then incubating bio-Aβ42 through the same catheter. Amytrapper catheters were blocked with 0.01% BSA in PBS (Sigma) overnight at 4^0^C. BSA was removed and catheters were washed 5 times with PBS. 200 ng of Aβ42 (Anaspec, CA) was mixed in 350 μl of human serum, which was then transferred into the coated catheter and incubated for 2 hours. After three washes with PBS, another 350 μl aliquots of human serum containing 40 ng of bio-Aβ42 was incubated in the catheter for 2 hours, and the evaluation of binding was performed as previously stated.

## Results

We have been working on the development of a peptide-based compound, Amytrap, for amyloid reduction in circulation. Amytrap has been shown to bind Aβ42 *in vitro* and *in vivo* [12]. In a previous study we have shown that the device called Amytrapper column, containing the API (RI-peptide) coupled to sepharose beads, binds and removes Aβ in sera or plasma spiked with known amounts of Aβ42. In the present study, the novel Amytrapper catheter (polycarbonate) containing the coated API (RI-peptide) was tested in buffer or human serum spiked with known amounts of bio-Aβ42 or AD patient plasma to confirm the removal of Aβ specifically. The Amytrapper catheter performed as expected by specifically trapping bio-Aβ42 or native Aβ from all the test samples.

### Synthesis and functional characterization of RI-Mal-PEG4 conjugated peptide and Amytrapper catheter

**Figure 1:**
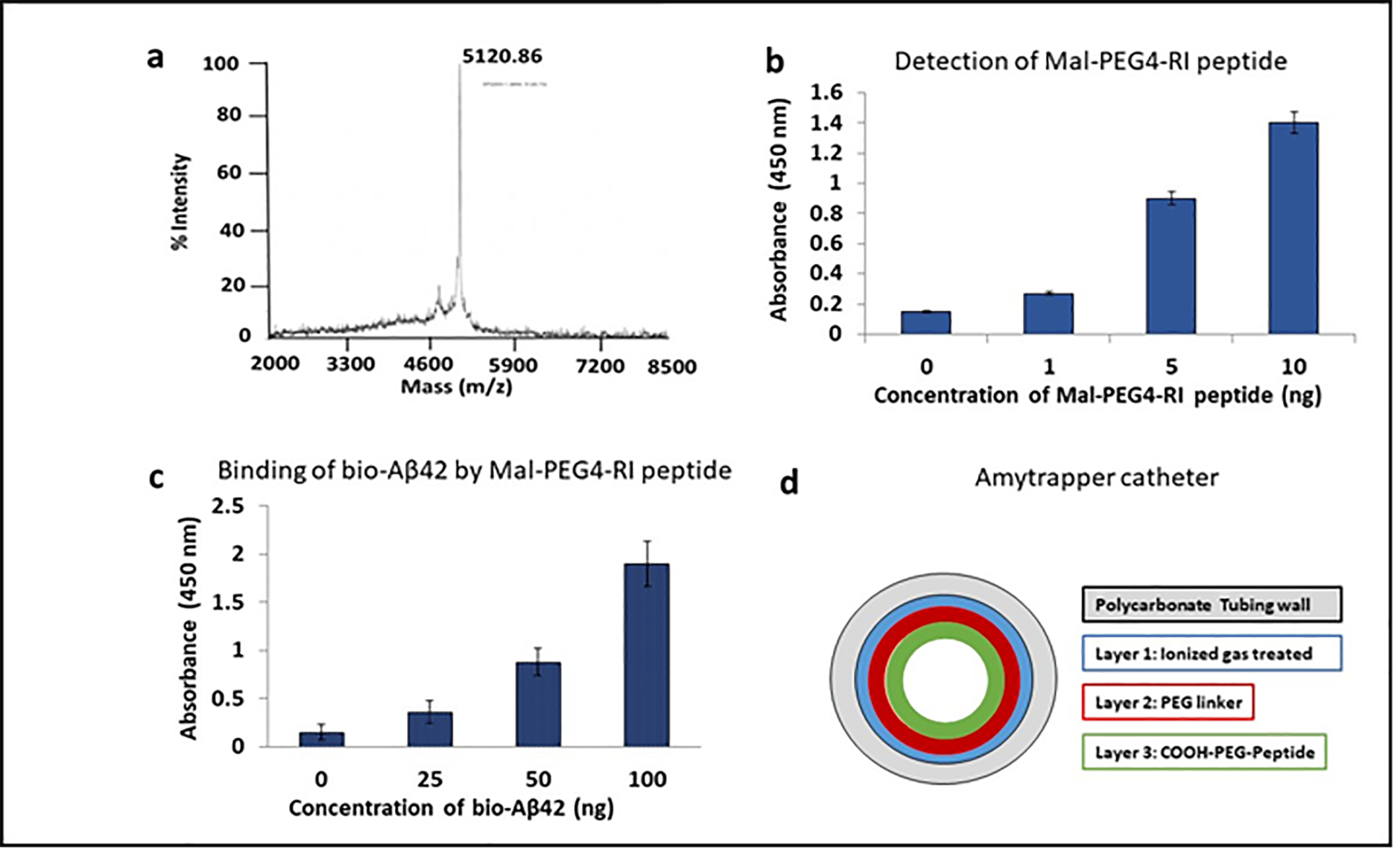
Functional validation of RI-Mal-PEG4 conjugated peptide and generation of Amytrapper Catheter. **a** The detection of conjugate of Tetrameric RI peptide with Mal-PEG4 acid as purified and assayed by LC-MS. **b** Conjugation was confirmed by ELISA detection of 1, 5 or 10 ng of conjugated tetramer using a rabbit anti-tetramer antibody. **c** ELISA showing RI-peptide-PEG conjugate retains its binding of Aβ42. 100ng of RI tetramer conjugated peptide was coated and probed with 0, 25, 50, or 100 ng/well of biotinylated Aβ42. Values are expressed mean absorbance ± SD is plotted. **d** Schematic diagram of Amytrapper catheter coated with RI-Mal-PEG4 conjugated peptide.

Tetramic RI peptide (MW 4793, Lifetein, NJ) was conjugated with Mal-PEG4-COOH followed by purification and analysis by LC-MS. After conjugation, the MW of the RI peptide was found to be 5,120 daltons (Figure 1a). The successful conjugation was further confirmed by an ELISA using Rabbit anti Tetramer antibody [12]. Graded concentrations (0, 1, 5, 10 ng) of conjugated peptide were tested. The gradation in specific binding absorbance was observed with increasing concentration of conjgated Tetramic RI peptide (Figure 1b). The conjugated peptide was then tested for binding to bio-Aβ42 by ELISA as described in methods section. The binding of bio-Aβ42 showed specific and concentration dependent binding by the RI conjugated peptide (Figure 1c).

### Coating of RI peptide (Amytrap peptide) on polycarbonate catheter

Coating of RI-Mal-PEG4 conjugated peptide to polycarbonate catheter was performed by utilizing multiple steps by Formacoat (Chaska, MN), as described in methods section. This process generated Amytrapper catheter. The schematic diagram of the coated layers on the catheter is shown in Figure 1d. The total Tetramic RI peptide coated internally on the the catheter is 4 μg/cm of length as quantified by using Pierce™ BCA Protein Assay Kit (Thermo Scientific, USA).

### Binding of biotinylated Aβ42 (bio-Aβ42) to API coated catheter (Amytrapper catheter)

**Figure 2:**
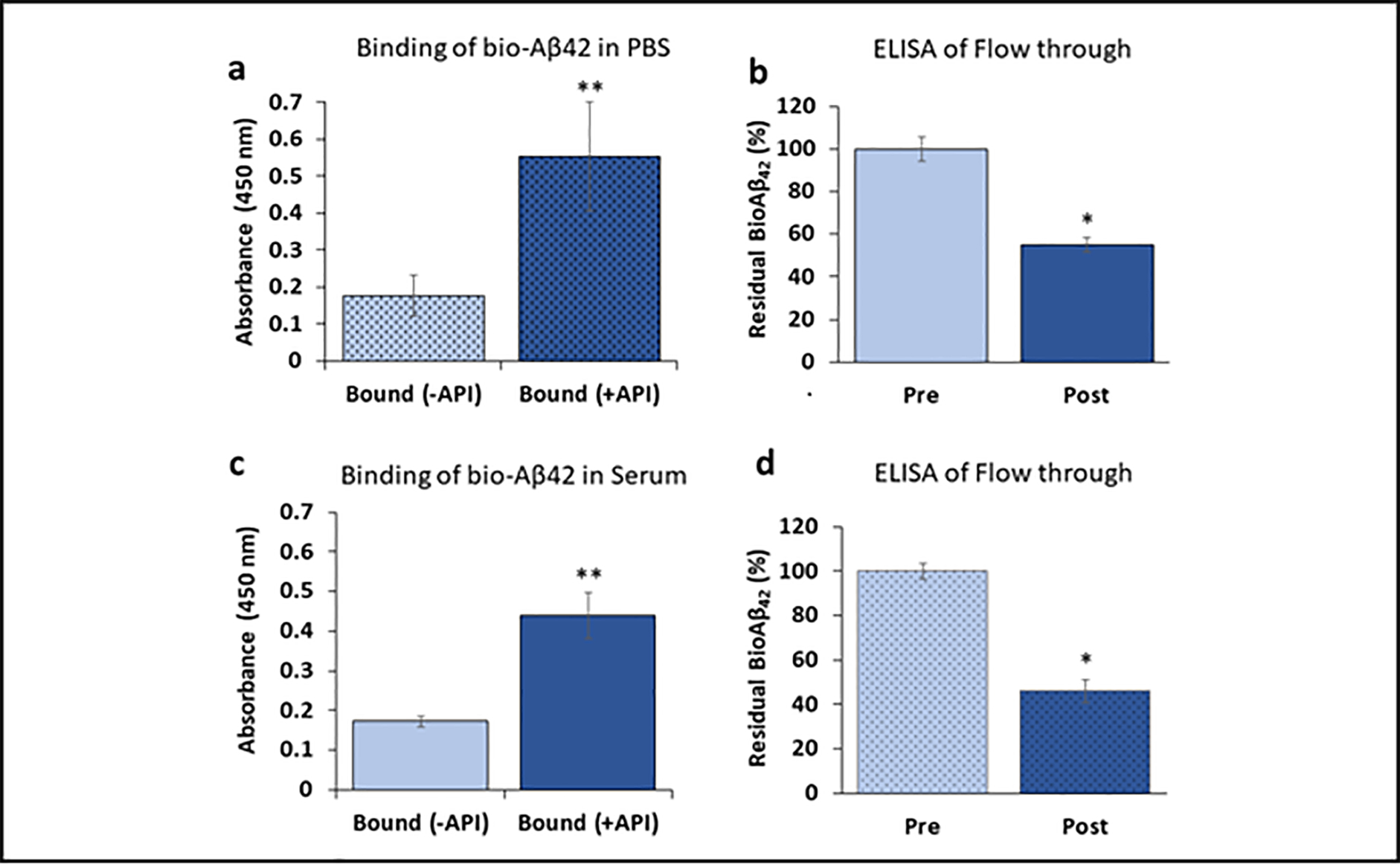
Binding of bio-Aβ42 to API coated catheter. **a** Binding and **b** Elisa of API coated catheter using PBS spiked with bio-Aβ42. **c** Binding and **d** Elisa of API coated catheter using human serum spiked with bio-Aβ42. Elisa data were collected from pre and post catheter fractions. Values expressed as absorbance in binding and percentage in flow through Elisa are represented as Mean ± SD from triplicate measurements. Student’s *T*-test was used to determine significance. *p<0.01, **p<0.001

Functional properties of Amytrapper catheter were examined by specific assays. Aβ binding was performed in PBS buffer before testing in human serum. Bio-Aβ42 was mixed in PBS and incubated in the catheters. Binding results for bio-Aβ42 in PBS from Amytrapper catheter were observed to be more than 200 percent higher as compared to non-coated catheter (Figure 2a). ELISA was performed on PBS samples before and after incubation in catheters. There was a 45% reduction in the total levels of bio-Aβ42 in the post Amytrapper catheter flow through PBS samples (Figure 2b). Results show that Amytrapper catheter incubated with human serum spiked with bio-Aβ42 has 150 percent increased Aβ binding as compared to non-coated catheter (Figure 2c). ELISA was also performed on serum samples before and after incubation inside the catheter. There was a 58% reduction in the levels of total bio-Aβ42 in the post Amytrapper catheter flow through serum samples (Figure 2d). Thus, the amount of bio-Aβ42 retained on the catheter is consistent with the reduction in the flow through.

### Binding of Aβ from plasma of AD patients by Amytrapper matrix or catheter

**Figure 3:**
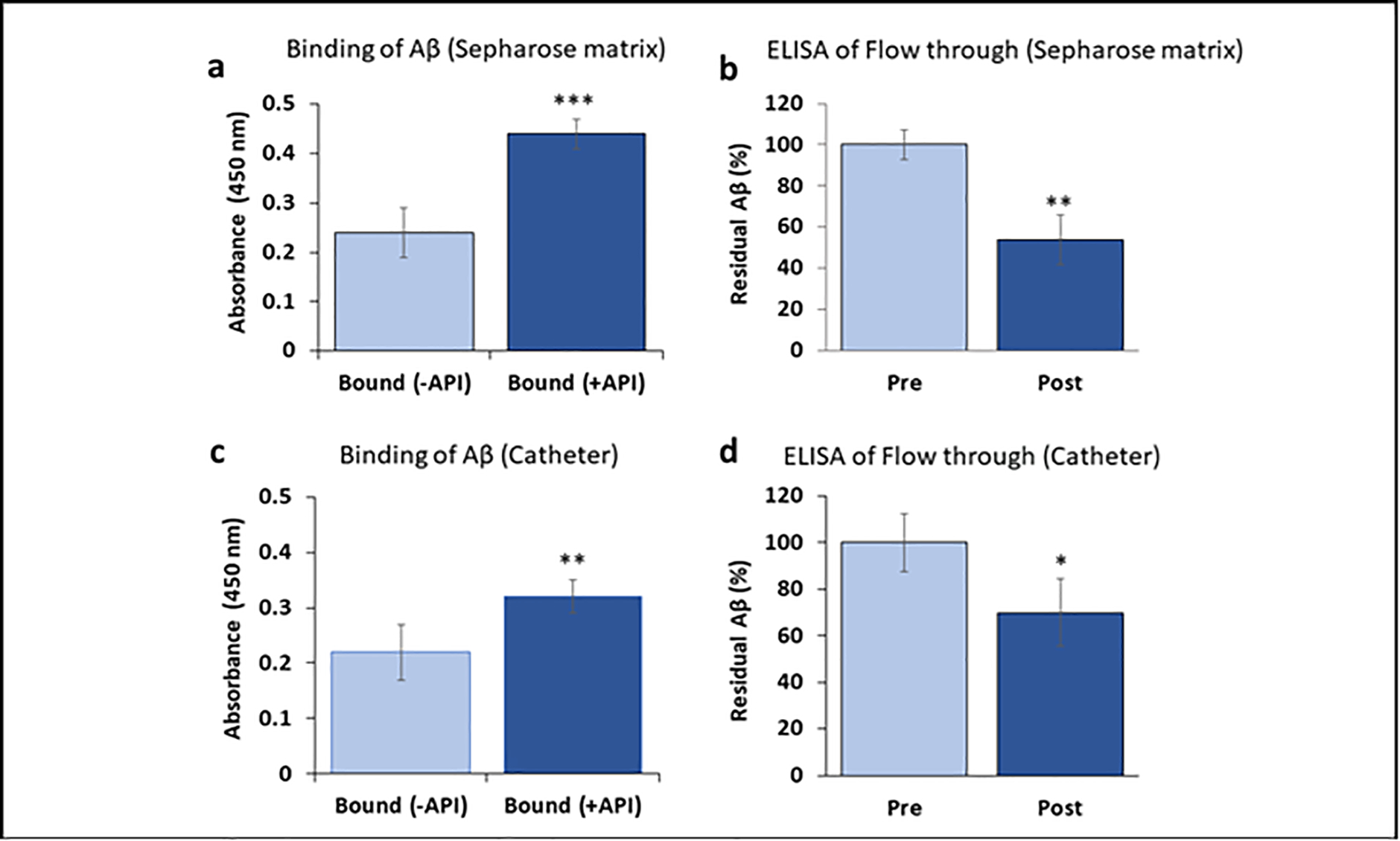
Binding of native Aβ in Alzheimer’s patient plasma to API coated Sepharose beads or catheter. **a** and **c** are binding data. Values expressed as absorbance in binding and percentage in flow through Elisa are represented as Mean ± SD from triplicate measurements. **b** and **d**, Elisa data were collected from pre and post beads or catheter fractions. Student’s *T*-test was used to determine significance. *p < 0.05, **p < 0.01,***p < 0.001

Amytrapper matrix has been shown to bind efficiently and specifically to bio-Aβ42 from spiked normal serum and plasma samples [12]. We wanted to test the native Aβ binding efficiency by Amytrapper matrix and Amytrapper catheter in plasma from AD patients. AD plasma samples were incubated with Amytrapper matrix or Amytrapper catheter. Total protein concentration of the sepharose beads was estimated to be 0.2 mg/ml following the method as described [12]. This represents that total peptide (API) concentration of 20 μg/100 μl beads exposed to plasma. Proportionate amount of API was exposed in 5 cm long catheter coated at 4 μg/cm. ELISA was performed on samples before and after incubation in order to evaluate this binding. The results showed a significant Aβ binding observed from RI-peptide beads exposed with AD plasma as compared to unconjugated matrix (Figure 3a). There was a 46% reduction in the levels of native Aβ in the AD plasma samples after incubation with Amytrapper matrices (Figure 3b). This observation further validated the binding efficacy of Amytrapper matrices for native Aβ. We then tested binding efficiency of Amytrapper catheter to native Aβ in plasma from AD patients. AD patient’s plasma was incubated in both control catheter and Amytrapper catheters. Aβ binding in Amytrapper catheter was observed to have increased around 100 percent compared to control catheter (Figure 3c). Consistently, on ELISA, there was 30% reduction of native Aβ in the AD plasma samples after incubation inside Amytrapper catheter (Figure 3d).

### Binding of Aβ42 from whole blood by Amytrapper catheter

**Figure 4:**
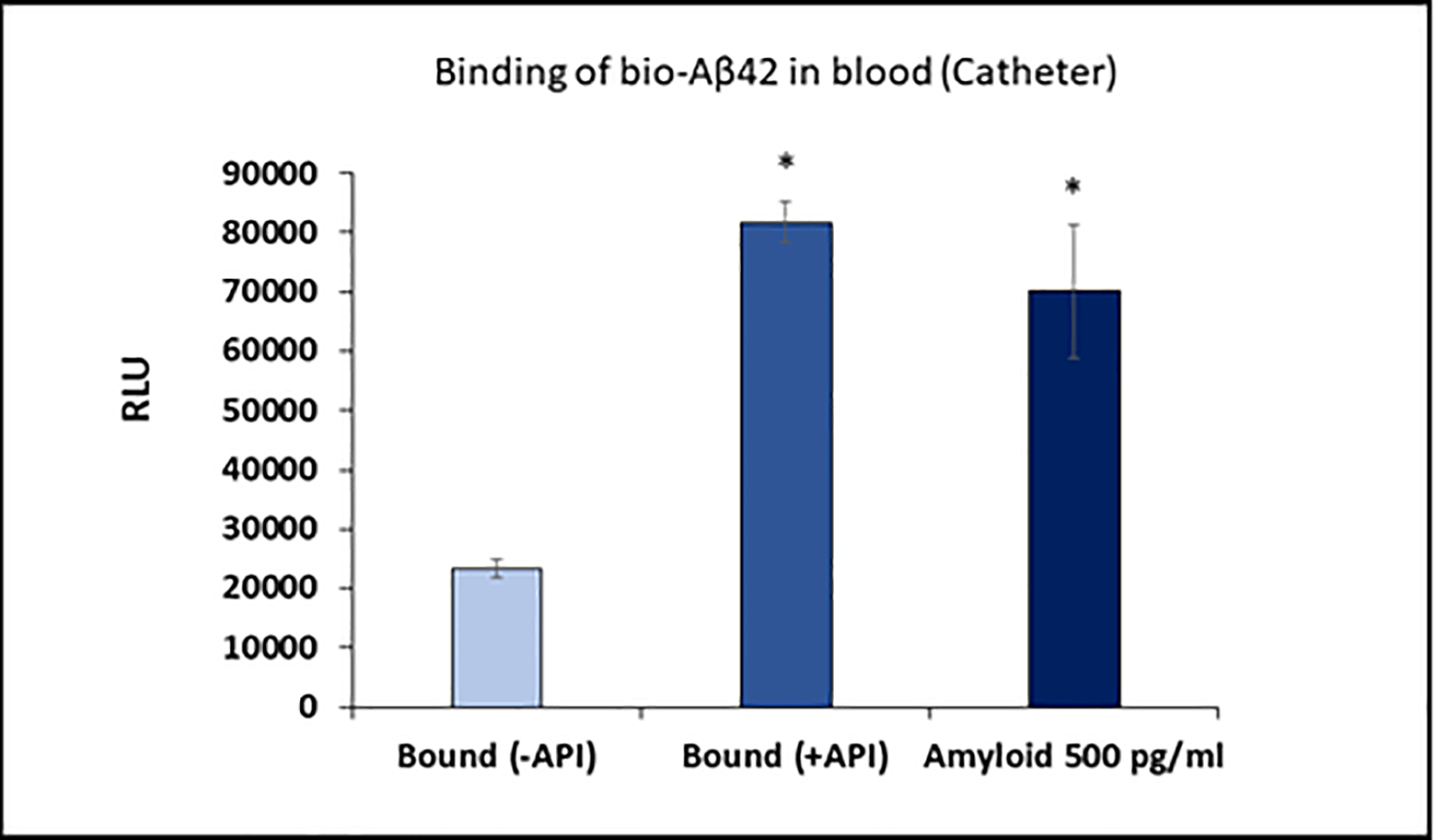
Binding of bio-Aβ42 to API coated catheter using human blood spiked with bio-Aβ42. Values are expressed in relative luminescence units (RLU) presented as Mean ± SD from triplicate measurements. RLU from standard bio-Aβ42 is shown in third bar. Student’s *T*-test was used to determine significance. *p<0.01

In order to improvise the function of the catheter device, we succeeded in a pilot attempt to run whole blood from humans following the methods as provided. Results showed more than 4-fold higher bio-Aβ42 binding in API versus non-API coated catheter (Figure 4). This binding efficiency captures nearly 500 pg/ml Aβ42 which is close to clinical value of total amyloid in human circulation. With direct and efficient Aβ binding from whole blood, these results show the success of our device design for eventual clinical use.

### Specificity of Amytrapper catheter

**Figure 5:**
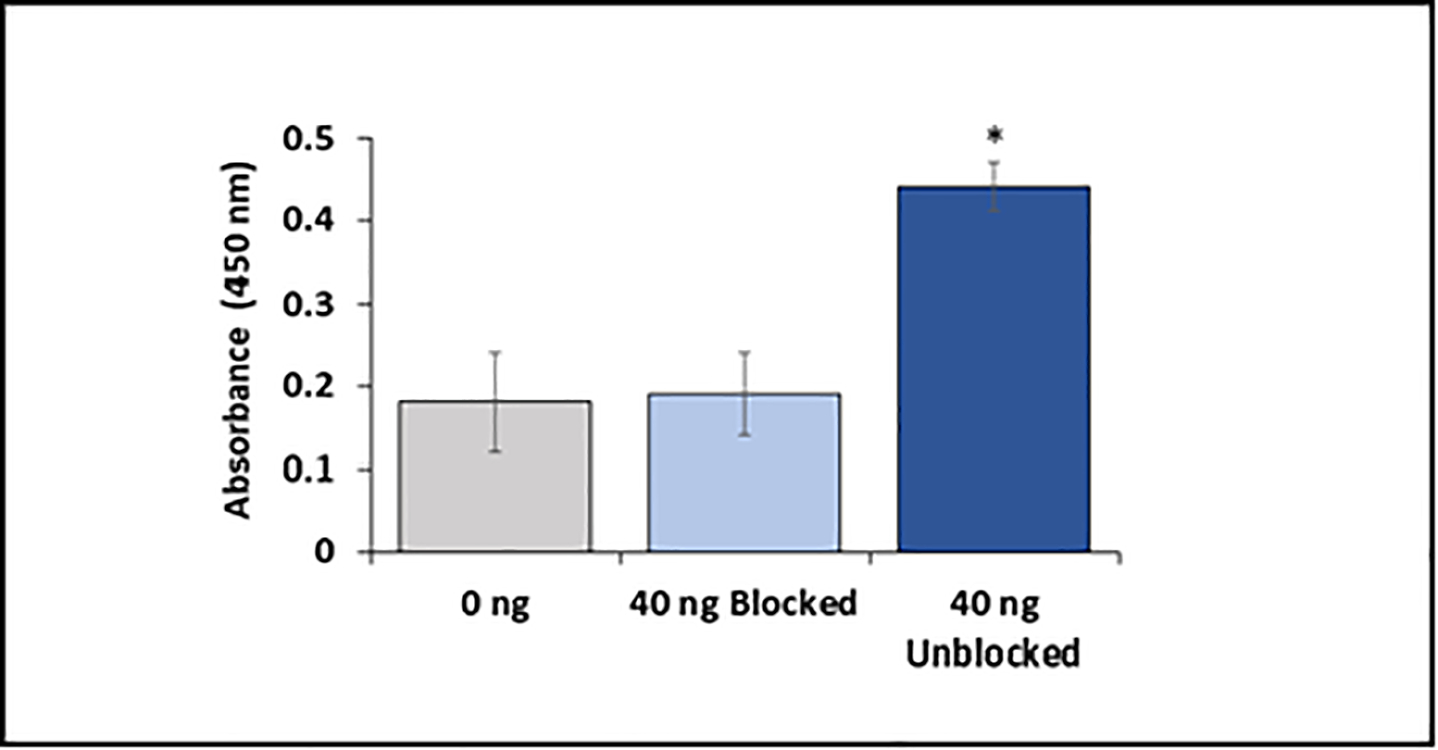
Specificity of Amytrapper catheter for Aβ42. Data are represented in the figure for blocked and unblocked samples. Values are represented as absorbance in binding and presented as Mean ± SD from duplicate measurements. Student’s *t*-test was used to determine significance. *p <0.001

In order to assess the Amytrapper catheter’s specificity for its target molecule, Aβ42, the Amytrapper catheters were saturated with 5-fold excess unlabeled Aβ42. The saturated catheters were then incubated with bio-Aβ42 as described in the methods. The results support that Amytrapper catheter presents a high specificity for Aβ42. Catheters blocked with unlabeled Aβ42 were calculated to have almost no difference in binding when compared with the control (0 ng bio-Aβ42; Figure 5). The unblocked catheters showed significant and specific 2.5-fold Aβ42 binding as shown in figure 5. This observation further validated the specificity of Amytrapper catheter for Aβ42.

## Discussion

Drug development for AD has proven to be difficult as both small molecules and immunotherapies have failed due to efficacy or toxicity reasons. Many of the current strategies aim to reduce Aβ-plaque burden by reducing blood Aβ levels. In addition to therapies, strenuous research efforts continue to develop effective disease-modifying treatments (DMTs) for AD to relieve symptoms. Extracorporeal system to remove blood Aβ has been shown to be a useful therapeutic strategy for AD. Extracorporeal blood Aβ-removal systems (E-BARS) using hollow membranes to remove Aβ oligomers have been developed [14,15]. The blood of patients was introduced to a plasma separator and the separated plasma was introduced to a device for the removal of Aβ oligomers. The device was composed of hollow-fiber membranes with appropriate larger pore sizes than hemodialyzers. Aβ oligomers of large molecular weights hardly passed through the pores, and thus retained in the hollow fibers, and were discarded in waste plasma. Smaller molecules, including albumin, can pass through the pores and return to the patients. A prospective study of patients undergoing hemodialysis showed that cognitive functions were maintained or slightly improved, and it appears that a major mechanism of Aβ’s removal by hollow fiber dialyzers might be through adsorption [16]. This approach captures larger molecules in plasma. These observations prompted us to investigate a similar extracorporeal system employing our proprietary API coated matrix or catheter to remove Aβ42.

As previously demonstrated by our group, the Amytrap-peptide has shown effective binding of Aβ42 *in vitro* and it reduced Aβ42 levels in a transgenic mice model [12]. Amytrap has been shown to be safe, non-immunogenic, non-toxic and highly stable [12,13]. PEG coatings have been widely used in medical devices because of their non-immunogenic and protein repellent properties [17,18]. We, therefore used a pegylated version of Amytrap-peptide as API to perform the current study. Promising results in the application of catheter-based drug techniques motivate continued investigation of this technology [19–23]. To that effect, the present study shows that the pegylated Amytrap peptide coated catheter (Amytrapper catheter), examined as a tool for DMT, is able to bind and retain synthetic and native Aβ42. The results reveal that the Amytrapper catheter has the potential to become a novel disease modifying therapy.

The affinity between Albumin and Aβ has given rise to AMBAR (Alzheimer’s Disease Management by Albumin Replacement) project for steps towards AD treatments. Replacement of endogenous albumin with 5% Human Albumin through Plasma Exchange (PE) schedules have been shown to be promising DMT [24,25]. The Amytrapper catheter being evaluated in the current study is a tool which sequesters both bio-Aβ42 spiked in serum and endogenous Aβ in AD patients’ plasma by utilizing more specific peptide interactions for its target Aβ compared to albumin and Aβ. This approach seems superior in that it does not require exogenous addition of albumin and Aβ levels are directly reduced by affinity binding with API in catheter while preserving native consistency of patient plasma. This qualifies Amytrapper catheter as an enhanced *ex* vivo DMT tool to be evaluated.

We had earlier demonstrated that the Amytrap peptide as Amytrapper matrix (on Sepharose beads) binds to Aβ protein present in human serum with robust efficiency and specificity [13]. The current study, compared the target binding efficacies of Amytrapper matrix and Amytrapper catheter. Under comparable experimental conditions, using the same AD patient plasma on both matrices and catheters, we found Amytrapper catheter binds to native Aβ with approximately 15% less efficiency than Amytrapper matrix when quantitated through immunoassay. These conditions warrant further modulations of flow rate of plasma to enhance sufficient contact time for native Aβ towards surface bound API. In addition to these observations, we were able to show efficient binding of Aβ42 on Amytrapper catheter from whole blood spiked with bio-Aβ42. This improvised execution plan has positive ramifications towards skipping plasma separation steps in clinical use of the Amytrapper devices. With direct and efficient Aβ binding from whole blood, we are one step closer in our prototype device design.

In order to show that retention of Aβ42 by Amytrapper catheter was specific to Aβ42, binding sites on the Amytrapper catheter were pre-blocked with excess Aβ42 followed by binding experiment utilizing human serum spiked with bio-Aβ42. If the binding were specific, then the non-biotinylated Aβ42 would block the binding sites, and therefore prevent the eventual binding of bio-Aβ42. As shown in the results, unlabeled Aβ42 prevented eventual binding of bio-Aβ42 to Amytrapper catheter, demonstrating that the retention of Aβ42 by Amytrapper was indeed specific to Aβ42. The specificity of Amytrapper catheter is consistent with other studies on RI-peptides, which have shown that RI-peptides have high selectivity for their target and are thus unlikely to cross-react with other proteins [13,26].

In summary, the present study demonstrates that the Amytrapper catheter effectively captures circulating Aβ. Results further confirm that this binding is highly specific to Aβ. Overall, the results show that Amytrapper catheter is functional in binding Aβ with high affinity, indicating that it is ready to move forward to *in vivo* studies. Further investigations on the Amytrapper catheter to confirm its efficiency on amyloid capture under more clinically relevant experimental set up (for instance, structured flow of plasma/blood through catheter) are warranted.

## Conclusion

Extracorporeal system to remove blood Aβ may serve as a Disease-modifying treatment (DMT) for Alzheimer’s Disease (AD). The current study successfully demonstrates the development and evaluation of amyloid beta (Aβ) sequestering peptide (Amytrap) based Amytrapper catheter, which specifically binds and clears out Aβ from spiked normal serum or AD patient serum. Results encourage additional research efforts toward viable clinical development of high affinity and highly specific DMT for AD.

## Data Availability

The datasets generated during and/or analyzed during the current study are available from the corresponding author on reasonable request.
